# The association between peripheral inflammation, brain glutamate and antipsychotic response in Schizophrenia: Data from the STRATA collaboration

**DOI:** 10.1016/j.bbi.2023.05.005

**Published:** 2023-05-12

**Authors:** Sunniva Fenn-Moltu, Bill Deakin, Richard Drake, Oliver D. Howes, Stephen M. Lawrie, Shôn Lewis, Naghmeh Nikkheslat, James T.R. Walters, James H. MacCabe, Valeria Mondelli, Alice Egerton

**Affiliations:** a Department of Psychosis Studies, Institute of Psychiatry, Psychology & Neuroscience, King’s College London, London, UK; b Department of Forensic and Neurodevelopmental Sciences, Institute of Psychiatry, Psychology and Neuroscience, King’s College London, London, UK; c Centre for the Developing Brain, School of Biomedical Engineering & Imaging Sciences, King’s College London, London, UK; d Division of Neuroscience and Experimental Psychology, School of Biological Sciences, Faculty of Biology, Medicine and Health, University of Manchester, Manchester, UK; e National Institute for Health Research (NIHR) Mental Health Biomedical Research Centre, South London and Maudsley NHS Foundation Trust, King’s College London, London, UK; f Psychiatric Imaging Group, MRC London Institute of Medical Sciences, Hammersmith Hospital, London, UK; g Division of Psychiatry, University of Edinburgh, Edinburgh, UK; h Division of Psychology and Mental Health, School of Biological Sciences, Faculty of Biology, Medicine and Health, University of Manchester, Manchester, UK; i Department of Psychological Medicine, Institute of Psychiatry, Psychology and Neuroscience, King’s College London, London, UK; j MRC Centre for Neuropsychiatric Genetics and Genomics, Division of Psychological Medicine and Clinical Neurosciences, School of Medicine, Cardiff University, Cardiff, UK

**Keywords:** ^1^H-MRS, Cytokines, Glutamate, Inflammation, Antipsychotic response, Schizophrenia

## Abstract

Glutamate and increased inflammation have been separately implicated in the pathophysiology of schizophrenia and the extent of clinical response to antipsychotic treatment. Despite the mechanistic links between pro-inflammatory and glutamatergic pathways, the relationships between peripheral inflammatory markers and brain glutamate in schizophrenia have not yet been investigated. In this study, we tested the hypothesis that peripheral levels of pro-inflammatory cytokines would be positively associated with brain glutamate levels in schizophrenia. Secondary analyses determined whether this relationship differed according to antipsychotic treatment response. The sample consisted of 79 patients with schizophrenia, of whom 40 were rated as anti-psychotic responders and 39 as antipsychotic non-responders. Brain glutamate levels were assessed in the anterior cingulate cortex (ACC) and caudate using proton magnetic resonance spectroscopy (^1^H-MRS) and blood samples were collected for cytokine assay on the same study visit (IL-6, IL-8, IL-10, TNF-α and IFN-γ). Across the whole patient sample, there was a positive relationship between interferon-gamma (IFN-γ) and caudate glutamate levels (r = 0.31, p = 0.02). In the antipsychotic non-responsive group only, there was a positive relationship between interleukin-8 (IL-8) and caudate glutamate (r = 0.46, p = 0.01). These findings provide evidence to link specific peripheral inflammatory markers and caudate glutamate in schizophrenia and may suggest that this relationship is most marked in patients who show a poor response to antipsychotic treatment.

## 1 Introduction

Mounting evidence suggests that both dysfunction of glutamate neurotransmission and immune system alterations may contribute to the aetiology of schizophrenia, with substantial bodies of support emerging for both theories (Baumeister et al., 2014; Beck et al., 2016; Benrós and Mortensen, 2015; Egerton et al., 2020; Meyer, 2014). It is also becoming clear that these pathological mechanisms may be linked, as an excessive activation of immune pathway may dysregulate glutamate concentrations, leading to behavioural alterations (Haroon et al., 2017; Miller et al., 2016). Most empirical research investigating this link between inflammation and glutamate dysfunction has been conducted in subjects with mood disorders (Haroon et al., 2017; Miller et al., 2016), whilst work on glutamate neurotransmission and immune system alternations in schizophrenia have largely proceeded along separate paths. Given the emerging relevance of both the glutamate and immune systems to the onset and treatment-responsivity of schizophrenia (Egerton et al., 2012, 2020, 2021, 2022; He et al., 2020; Iwata et al., 2019; Lin et al., 1998; Mondelli et al., 2015; Mouchlianitis et al., 2016; Nakahara et al., 2021; Tarumi et al., 2020; Zhang et al., 2004), it is important to understand the relationship between inflammation and glutamate in this condition.

The glutamate hypothesis of schizophrenia posits that increased activation of pyramidal glutamatergic neurons is caused by disinhibition of NMDA-regulated GABAergic inhibitory interneurons due to NMDA receptor hypofunction (Homayoun and Moghaddam, 2007; Lisman et al., 2008; Olney et al., 1999; Steiner et al., 2012). The concentration of glutamate in the human brain can be measured using proton magnetic resonance spectroscopy (^1^H-MRS). ^1^H-MRS *meta*-analyses find that, overall, in schizophrenia, glutamate metabolites may be elevated in the basal ganglia and reduced in the medial frontal cortex (mFC, including anterior cingulate cortex, ACC) (Marsman et al., 2013; Merritt et al., 2016, 2022; Nakahara et al., 2021; Smucny et al., 2021; Sydnor and Roalf, 2020). However, the extent and direction of glutamate abnormality may relate to illness stage, severity, medication effects, genetic and other factors (Bustillo et al., 2017; Merritt et al., 2016, 2021, 2022; Nakahara et al., 2021). Of particular interest are findings from some studies indicating that mFC/ACC glutamate metabolites may be increased in patients who show a poor compared to good response to antipsychotic treatment (Egerton et al., 2012, 2018, 2021; Iwata et al., 2019; Mouchlianitis et al., 2016; Szulc et al., 2013; Tarumi et al., 2020). Overall, glutamate levels are more variable in schizophrenia than in healthy volunteers (Merritt et al., 2022). This could indicate differential influences of contributing mechanisms on glutamate levels, potentially including inflammation, which could also relate to treatment response.

Epidemiological studies have recognised infections and autoimmune diseases as risk factors for developing schizophrenia (Benrós and Mortensen, 2015; Canetta and Brown, 2012; Cullen et al., 2019), and immune-related gene polymorphisms have been associated with the disorder (Hudson and Miller, 2018). Altered levels of circulating pro- and anti-inflammatory cytokines in schizophrenia provide more direct evidence for immune dysfunction, with increased levels of proinflammatory cytokines present in the peripheral plasma and serum of prodromal (Stojanovic et al., 2014), first episode (Di Nicola et al., 2013; Zajkowska and Mondelli, 2014), acutely ill (Goldsmith et al., 2016) and chronic (Coelho et al., 2008; Miller et al., 2011) schizophrenia. Moreover, high levels of pro-inflammatory cytokines have also been associated with more severe symptoms at disease onset and after administration of antipsychotics (Chase et al., 2016; Frydecka et al., 2014; Lee et al., 2017; Miller et al., 2011; Stojanovic et al., 2014), and with worse antipsychotic response (Lin et al., 1998; Mondelli et al., 2015).

The convergence of inflammation and the glutamate system has most thoroughly been investigated in the context of major depressive disorder. For example, interferon-alpha, a common treatment for hepatitis C, increases glutamate concentration in the caudate and ACC, and this correlates with the development of depressive symptoms (Haroon et al., 2014). Higher levels of peripheral inflammatory markers also predict worse antidepressant efficacy of the glutamate antagonist ketamine in individuals with major depressive disorder (Hashimoto, 2015; Walker et al., 2015). Furthermore, increased plasma levels of C-reactive protein (CRP), a nonspecific marker of inflammatory processes, has been associated with increased levels of glutamate in the caudate of individuals with depression (Haroon et al., 2016). Higher levels of IL-6 has also been associated with higher concentrations of glutamate in the dorsal ACC in adolescents with depression (Ho et al., 2021). There is thus evidence that inflammation may increase brain glutamate metabolites in patients with mood disorders and contribute to treatment resistance (Miller and Raison, 2016), but it is unknown whether this relationship is also observed in schizophrenia.

The primary aim of the current study is to determine whether peripheral cytokine levels are related to brain glutamate in patients with schizophrenia. We hypothesised that markers of increased peripheral inflammation would be associated with increased levels of brain glutamate. Secondly, given that both glutamate increases and peripheral inflammation may be more pronounced in antipsychotic non-responsive schizophrenia and that inflammation has been suggested to lead to treatment resistance depression through its effects on the glutamatergic system (Haroon and Miller, 2017), we hypothesised that the relationship between peripheral inflammation and brain glutamate levels would be stronger in antipsychotic non-responsive compared to antipsychotic responsive illness, reflecting greater activation of inflammatory-glutamate pathways.

## 2 Methods

### 2.1 Regulatory approvals

The study was approved by the NHS Research Ethics Committee (ref 15/LO/0038). All participants provided written informed consent.

### 2.2 Participants

Recruitment and assessment took place at King’s College London and Universities of Manchester, Edinburgh and Cardiff. Participants were 18–65 years of age and had DSM-5 diagnosis of schizophrenia or schizophreniform disorder and were able to understand and consent to the study procedures. Exclusion criteria were pregnancy, severe head injury, meeting ICD criteria for substance misuse or psychotic disorder secondary to substance misuse, treatment with clozapine in the last 3 months, or contraindications to MRI. Clinical diagnosis was confirmed using the MINI (Sheehan et al., 1998), and illness severity assessed using the Positive and Negative Syndrome Scale (Kay et al., 1987) and Clinical Global Impression scale for Schizophrenia (Haro et al., 2003).

### 2.3 Definition of antipsychotic responder & non-responder groups

Antipsychotic responders and antipsychotic non-responders were defined as described in Egerton et al. (2021). Briefly, antipsychotic responders (R) were defined as having had (1) treatment with only 1 antipsychotic drug since illness onset, or, if there were any treatment changes, then these were due to adverse effects as opposed to non-response; (2) a CGI-SCH severity score of < 4; (3) a PANSS total score of < 60 (Leucht et al., 2005); and (4) a compliance rating scale (CRS) score (Kemp et al., 1996) of > 3. Antipsychotic non-responders (NR) were defined as having (1) documented treatment with at least 2 anti-psychotics for > 4 weeks each, at doses above the minimum therapeutic doses as defined by the British National Formulary; (2) a CGI-SCH severity score of > 3; (3) a PANSS total score of at least 70; and (4) a CRS of > 3.

### 2.4 Proton magnetic resonance spectroscopy

Glutamate levels were measured using ^1^H-MRS at 3 Tesla on either a General Electric MR750 (Chicago, USA), Philip Achieva (Philips Healthcare, The Netherlands) or a Siemens Verio magnetic resonance system, as previously described (Egerton et al., 2021). Sagittal T-1 weighted images were acquired to guide voxel positioning. Non-rotated voxels measuring 20 × 20 × 20 mm was positioned in the anterior cingulate cortex (ACC), and in the caudate. Further details and images of voxel positioning and spectral quality are provided in Egerton et al. (2021). Point RESolved Spectroscopy (PRESS, TE = 35msec; TR = 2000msec; 128 averages, bandwidth/sample frequency ± 2500 Hz, number of complex points = 4096) or the standard GE PROBE (PROton Brain Examination) sequence were used to acquire the ^1^H-MRS spectra. Additionally, unsuppressed water spectra were acquired. The spectra were analysed in LC Model (v.6.3-1L) using a standard LC Model basis set acquired using PRESS at 3 Tesla and a TE of 35 msec. Glutamate estimates were water-referenced, and the ^1^H-MRS voxels were also co-registered to the T1-weighted image to determine the voxel grey matter, white matter, and CSF fractions using Gannet software (version 2.0, https://www.gabamrs.com/). Glutamate values were then corrected for voxel tissue content: Mcorr = M*(wm + 1.21*gm + 1.55*CSF)/(wm + gm), M being the uncorrected metabolite, and wm, gm and CSF indicating the white and grey matter and CSF content (Gasparovic et al., 2006; Kreis et al., 1993).

Quality of ^1^H-MRS was determined by a review of LCModel estimates of spectral line width and signal-to-noise ratio. Spectra were excluded under any of the following criteria (1) absence of corresponding unsuppressed water acquisition; (2) compared with the overall mean for the voxel across all sites and participants, spectral line width was 2 standard deviations above; or (3) spectral signal-to-noise ratio was 2 standard deviations below. Individual metabolite concentration estimates associated with Cram´er Rao lower bounds (CRLB) > 20% were excluded. The primary outcome variable was glutamate. For completeness, data for glutamate plus glutamine (Glx), are presented in the (Supplementary Material (Supplementary Fig. 1, Supplementary Table 1).

### 2.5 Blood plasma assays

Plasma cytokine levels were measured as described in Enache et al. (2021). Venous blood samples were collected and centrifuged within one hour at 1300–2000g for 10 min. Cytokines were measured in duplicate using Meso Scale Discovery (MSD) V-plex immunoassays (MSD, Maryland, USA) according to the standard protocol provided by MSD. The standard Pro-inflammatory Panel 1 (human) kit was used for the measurement of IFN-γ, IL-1β, IL-2, IL-4, IL-6, IL-8, IL-10, IL-12p70, IL-13, and TNF-α. For the purpose of the analyses of this paper, we included only IL-6, IL-8, IL-10, TNF-α and IFN-γ as these were the cytokines which have been more consistently reported to be relevant for onset and treatment response in schizophrenia (Di Nicola et al., 2013; Enache et al., 2021; Kose et al., 2021; Mondelli et al., 2015; Pillinger et al., 2019).

### 2.6 Statistical analysis

Data were analysed using RStudio version 1.2.1335 (R Core Team, 2020) using the packages ppcor (Kim, 2015) and cocor (Diedenhofen and Muscg, 2015). Figures were made using ggplot2 (Wickham, 2016), corrplot (Wei and Simko, 2017), RColorBrewer (Neuwirth, 2014) and viridis (Garnier et al., 2021). Logarithmic transformation was applied to all plasma cytokines to normalise distribution before statistical analyses. Due to site effects, ^1^H-MRS metabolite concentration estimates were converted to Z-scores, calculated by subtracting the site mean from individual values, before dividing by the site standard deviation. Partial correlations tested the association between cytokine and glutamate levels in the ACC and caudate. All partial correlations were corrected for BMI, age, sex and smoking status. Partial correlations were also run including antipsychotic chlorpromazine equivalents (CPZE) as an additional covariate. To control for multiple comparisons, a Benjamini and Hochberg approach was employed with a false discovery rate (FDR) set at 0.25. To test whether the strength of relationship between the inflammatory markers which were associated with brain glutamate differed as a function of antipsychotic response, the strength of the correlation coefficients were compared between the groups using Fisher’s z-test and Zou’s confidence intervals, and general linear models were run to test for any interaction between the effects of antipsychotic response status and cytokine concentrations on brain glutamate, including the same covariates as stated above. This analysis was also carried out for IL-8, because in an overlapping sample of participants to those included in the current study, higher levels of IL-8 was associated with a poor response to antipsychotic treatment (Enache et al., 2021).

## 3 Results

### 3.1 Subject demographics

Eighty participants completed ^1^H-MRS imaging and blood cytokine assays (40 treatment responders, 40 treatment non-responders). One subject was excluded due to very high C-reactive protein levels (CRP > 50), likely indicative of acute infection/injury. Nine subjects were excluded due to missing BMI values. Of the remaining 70 participants, 5 were excluded from the caudate analysis due to the ^1^H-MRS data failing quality control, and 2 were excluded from the ACC analysis due to the ^1^H-MRS data failing quality control. The final numbers for analyses were 68 for ACC (36 R, 32 NR), and 65 for caudate (33 R, 32 NR). The final study population is a subset of the participants in Egerton et al. (2021) and Enache et al. (2021). Characteristics of the study population are shown in Table 1.

### 3.2 Relationships between inflammatory markers and brain glutamate

**Fig. 1:**
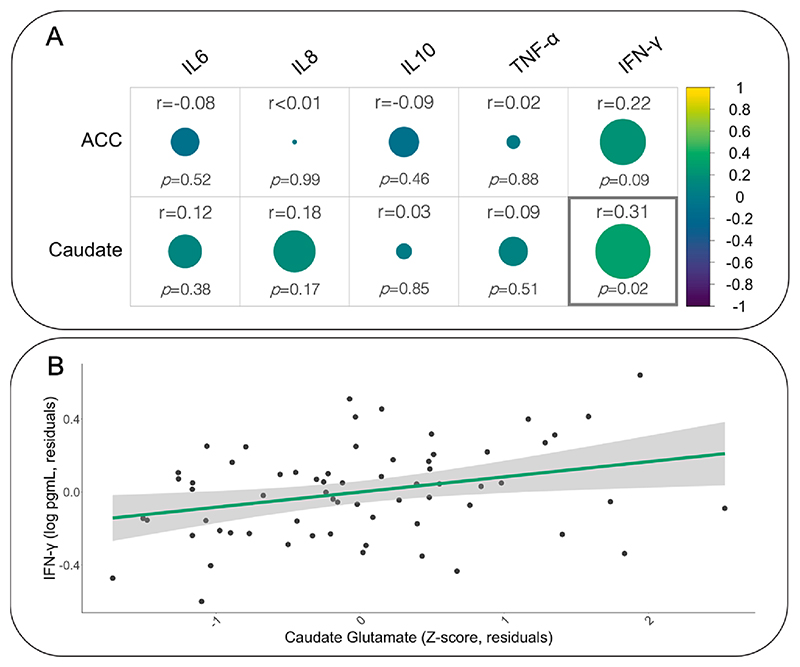
The association between peripheral cytokine levels and brain glutamate concentrations. A) Circle size represents the strength of the correlation, circle colour represents the strength and direction of the correlation. The association between peripheral IFN-γ and glutamate concentrations in the caudate is statistically significant (p = 0.02). B) Positive correlation between peripheral IFN-γ and glutamate concentration within the caudate are (r = 0.31, p = 0.02). The plot shows residuals corrected for age, sex, smoking status, and BMI.

Descriptive statistics are included in Table 2. Across all participants with schizophrenia, plasma IFN-γ concentration was positively correlated with glutamate concentrations in the caudate (r = 0.31, p = 0.02) Fig. 1. This relationship followed the same direction in the ACC but was only at trend level for significance (r = 0.22, p = 0.09). No other cytokines were significantly correlated with glutamate levels in the caudate or in the ACC. Covarying for CPZE did not meaningfully alter these results.

In this smaller subset of the participants reported in Egerton et al. (2021) and Enache et al. (2021), the group differences in ACC glutamate levels and IL-8 levels were non-significant.

### 3.3 Effect of antipsychotic response

**Fig. 2:**
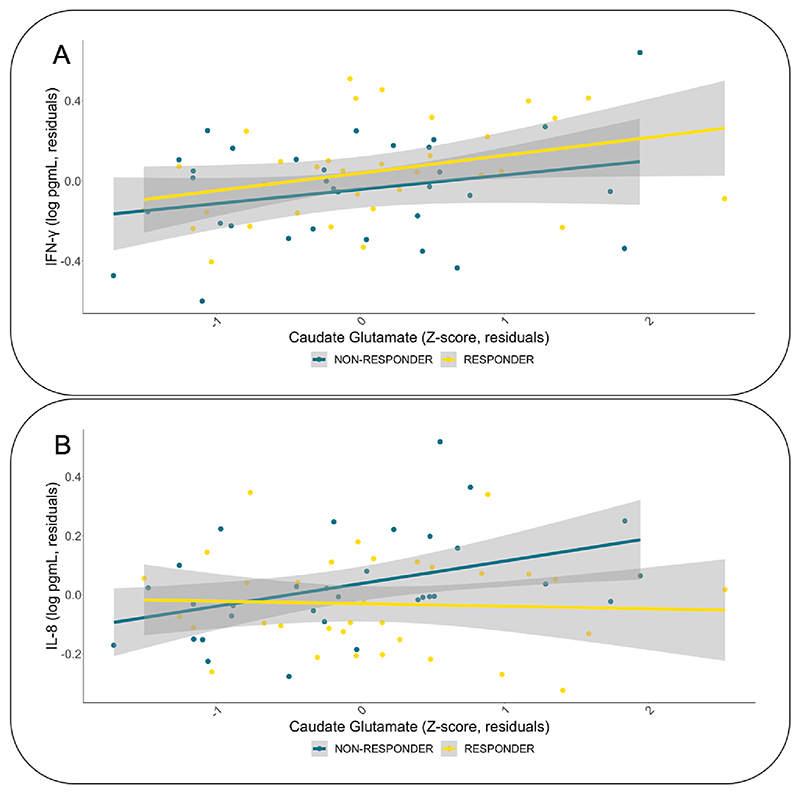
The association of IFN-γ (A) and IL-8 (B) with caudate glutamate concentrations according to antipsychotic response. The relationship between peripheral levels of IFN-γ and caudate glutamate concentration did not differ significantly between antipsychotic responders (yellow) and antipsychotic non-responders (blue). The relationship between peripheral levels of IL-8 and caudate glutamate concentration differed significantly between antipsychotic responders and antipsychotic non-responders. Plot showing residuals, corrected for age, sex, smoking status, and BMI.

The correlation coefficients between plasma IFN-γ concentration and caudate glutamate levels in antipsychotic responders (r = 0.25, p = 0.20) did not differ significantly from the correlation coefficient observed in antipsychotic non-responders (Fig. 2A, r = 0.18, p = 0.36): z = 0.2750, p = 0.783, CI: –0.4060 to 0.5351. There was also no group × IFN-γ interaction on caudate glutamate levels (β = –0.090, p = 0.85). The correlation coefficients between IL-8 concentrations and glutamate in the caudate differed significantly between the groups (Fig. 2B, z = −2.1895, p = 0.0286, CI: −0.9407 to 0.0532). There was a significant group × IL-8 interaction on caudate glutamate levels (β = −1.195, p = 0.048), with antipsychotic non-responders showing a positive correlation (r = 0.46, p = 0.01) not present in the antipsychotic responders (r = −0.07, p = 0.72). The groups did not differ significantly when looking at the association between IL-8 and glutamate in the ACC. Covarying for chlorpromazine equivalent dose did again not meaningfully alter these results.

## 4 Discussion

The primary aim of the current study was to test the hypotheses that markers of increased peripheral inflammation would be associated with increased levels of brain glutamate in schizophrenia. Our data showed a positive association between plasma IFN-γ levels and glutamate levels in the caudate, providing preliminary support for the convergence of immune and glutamatergic processes in schizophrenia. In the ACC, a similar pattern of association between IFN-γ and glutamate was observed, but below the threshold for statistical significance. Caudate or ACC glutamate levels were not associated with levels of the other analysed cytokines across the whole patient cohort. However, when assessing the antipsychotic responsive and antipsychotic non-responsive groups separately, peripheral levels of IL-8 and caudate glutamate concentrations were positively correlated in antipsychotic non-responders, but not in the antipsychotic responders. Together, these results provide initial evidence linking specific peripheral pro-inflammatory markers to caudate glutamate levels in schizophrenia and may suggest that these inflammatory-glutamatergic processes are most marked in those patients responding poorly to antipsychotic treatment.

Although previous studies have reported relationships between peripheral inflammation and other brain markers in schizophrenia, including associations with cortical thickness and volume (Jacomb et al., 2018; Kalmady et al., 2014; Mondelli et al., 2011; Wu et al., 2019; Zhang et al., 2016), neurocognitive impairment and psychomotor slowing (Goldsmith et al., 2020; Kogan et al., 2018; North et al., 2021), this is to our knowledge the first study to report a relationship between peripheral inflammation and brain glutamate in schizophrenia. Whilst inflammatory markers such as INF-α, CRP and IL-6 have been implicated in the interaction between inflammation and glutamate in depression (Felger et al., 2016; Haroon et al., 2014; Hashimoto, 2015; Ho et al., 2021; Walker et al., 2015), our main finding was a relationship between INF-γ and brain glutamate across the whole patient cohort, which could potentially suggest some degree of specificity to schizophrenia.

Whilst our study does not allow for inference about cause-and-effect relationships due to being cross-sectional, it has been established that peripheral immune activation can influence brain function through several pathways. As reviewed by Miller et al. (2013), the humoral pathways include circulating cytokines passing through leaky regions of the blood brain barrier, and active transport of circulating cytokines into the brain via cytokine specific transporters. The neural route involves activation of cytokine receptors on afferent nerve fibres that then transduce signals to the brain, and the cellular route is whereby chemokines released by activated microglia and adhesion molecules expressed in the central nervous system can attract activated peripheral cell types including monocytes and T cells to the meninges and brain parenchyma. Immune mediators have been shown to significantly influence the extracellular concentration of glutamate by altering the balance between glutamate release from glial and immune cells (Haroon et al., 2017), and its clearance mechanisms (McCullumsmith and Sanacora, 2015). Inflammatory cytokines and their signalling pathways can also activate the kynurenine (KYN) pathway of tryptophan metabolism, which generates neuroactive metabolites which can also affect glutamate metabolism (Chiappelli et al., 2018). Concurrently, both IFN-γ and IL-8 have been shown to modulate glutamatergic synaptic transmission in preclinical experiments (Cui et al., 2012; Garg et al., 2009); therefore, while the exact mechanistic pathways underlying the positive relationships between IFN-γ and caudate glutamate levels in the whole patient cohort and IL-8 in antipsychotic non-responders are not yet fully defined, these findings are consistent with evidence linking increases in peripheral proinflammatory cytokines with glutamate function.

As little is known about the interplay between peripheral inflammation and brain glutamate in schizophrenia, it is valuable to view our results within the context of studies assessing these factors independently. In an overlapping sample of participants to those included in the current study, higher levels of IL-8 (Enache et al., 2021) was associated with poor response to antipsychotic treatment. Previous research has also implicated IL-8 in the prognosis and therapeutic response of individuals with schizophrenia, with higher baseline IL-8 levels being associated with worse antipsychotic response (Zhang et al., 2004), and higher IL-8 may predicting less improvement in negative symptoms (He et al., 2020). Here we observed a positive relationship between IL-8 and caudate glutamate in the treatment non-responsive but not treatment responsive group. High serum levels of IFN-γ, alongside IL-6, have previously been reported to predict a poor response to antipsychotic medication after 12 weeks of treatment in patients with first episode psychosis (Mondelli et al., 2015), but we did not observe significant differences in the relationship between IFN-γ and brain glutamate between treatment responders and treatment non-responders in the current study. In an overlapping sample of participants to those included in the current study, higher levels of ACC glutamate (Egerton et al., 2021) were also associated with a poor response to antipsychotic treatment. Although caudate glutamate was not associated with antipsychotic response in this cohort (Egerton et al., 2021), some previous studies have found decreases in caudate glutamate during effective antipsychotic treatment (de la Fuente Sandoval et al., 2013; McQueen et al., 2021).

The relationship between IFN-γ and glutamate levels in the ACC showed a similar trend to that in the caudate, although it did not reach statistical significance. Compared to previous studies in depression finding associations between cytokines and dorsal ACC glutamate (Ho et al., 2021; Haroon et al., 2014) our ACC ^1^H-MRS voxel was positioned towards the more rostral perigenual ACC, and these regions may differ in both glutamate concentration and function (Li et al., 2022).

In the case of IL-8, we only observed a relationship with caudate glutamate in treatment non-responders, and there was no significant group difference between IL-8 and ACC glutamate, despite previous findings implicating ACC glutamate levels in treatment non-response (Egerton et al., 2012, 2018, 2021; Iwata et al., 2019; Mouchlianitis et al., 2016; Szulc et al., 2013; Tarumi et al., 2020). Previous research in depression has also emphasized the role of glutamate in the basal ganglia in the relationship with peripheral inflammation. For instance, Haroon et al. (2016) reported a significant association between peripheral inflammation and glutamate levels in the basal ganglia, but not in the ACC. In depression, the interplay between inflammation and the basal ganglia is suggested to be related to anhedonia and lack of motivation, and it is plausible that this rationale could extend to schizophrenia. Taken together, our results indicate a positive relationship between peripheral levels of IFN-γ and brain glutamate levels in schizophrenia, independent of antipsychotic response, whilst the relationship between IL-8 and caudate glutamate may be more specific to antipsychotic non-responsive illness.

Strengths of our study include the use of a standardised protocol to recruit a relatively large sample of patients across several sites in the UK. ^1^H-MRS acquisition sequences were harmonised across research sites, and metabolites estimated using the same analysis pipeline. We were also able to assay several cytokines previously implicated in schizophrenia. However, our study also has several limitations. As it was designed to investigate mechanisms underlying antipsychotic response in schizophrenia, it did not include a healthy control group. This means that we are unable to attribute the observed relationship between ^1^H-MRS glutamate measures and IFN-γ values to schizophrenia specifically. Further work is needed to assess the diagnostic specificity of this relationship, with comparison to both a healthy control group, and mood disorders such as MDD, where a relationship between brain glutamate levels and peripheral inflammation has previously been reported (Haroon et al., 2014, 2016; Ho et al., 2021). As clinical data were only gathered at a single cross-sectional timepoint, we did not ascertain the stability or timing of treatment response / non-response, and as non-response to antipsychotic medication was not determined prospectively and did not include an objective evidence of adherence, we are unable to determine the proportion of the NR group that would meet consensus guidelines for treatment-resistant schizophrenia (Howes et al., 2017). These factors may have contributed to a less distinct clinical separation between the R and NR groups and our ability to observe differences in inflammatory-glutamate relationships in relation to response status. Additionally, all participants were currently taking antipsychotic medication which may influence both glutamate levels (Egerton et al., 2017; Merritt et al., 2022; Zahid et al., 2022) and peripheral immune markers (Baumeister et al., 2016; Romeo et al., 2018; Tourjman et al., 2013). Future research could determine whether the relationships between IFN-γ, IL-8 and glutamate are present in medication-naïve psychosis and the association with subsequent antipsychotic response. As most of our patient sample were male (82%), we were not able to investigate potential effects of sex, and women may show greater responses to inflammatory challenges (Moieni et al., 2015). Lastly, ^1^H-MRS measurements cannot differentiate between intracellular and extracellular glutamate (Duarte and Xin, 2019). However, ^1^H-MRS-assessed glutamate levels correlate with transcranial magnetic stimulation-based measures of cortical excitability, suggesting that ^1^H-MRS measures reflect neural glutamatergic activity (Stagg et al., 2011).

In conclusion, the current study provides initial support for a positive relationship between peripheral inflammation and brain glutamate levels in schizophrenia. Further work is needed to clarify the mechanistic link between pro-inflammatory cytokines and brain glutamate concentrations, and to confirm whether inflammatory-glutamate mechanisms make a greater contribution to antipsychotic non-responsive schizophrenia.

## Supplementary Material

Supplementary Material

## Figures and Tables

**Table 1:** Subject Demographics and Clinical Details.

*Group*	Total	Responders (R)	Non-responders (NR)	*p*
	(n = 70)	(n = 37)	(n = 33)	(R vs NR)
***Age***	*29.54 (8.05)*	*29.89 (8.66)*	*29.15 (7.42)*	*0.999^b^*
***Male n***	*58 (82.86%)*	*31 (83.78%)*	*27 (81.82%)*	*0.828^c^*
**BMI**	*28.43 (4.87)*	*27.22 (4.89)*	*29.78 (4.55)*	***0.026^a^***
**Smoking currently n**	*41 (58.57%)*	*22 (59.46%)*	*19 (57.58%)*	*0.873^c^*
**Current Antipsychotic**				*0.231^c^*
Aripiprazole	*16*	*9*	*7*	
Olanzapine	*14*	*11*	*3*	
Risperidone	*10*	*6*	*4*	
Amisulpride	*6*	*2*	*4*	
Quetiapine	*7*	*1*	*6*	
Paliperidone	*4*	*1*	*3*	
Cloxipol	*3*	*2*	*1*	
Flupentixol	*2*	*1*	*1*	
Haloperidol	*1*	*1*	*0*	
Combination	*7*	*3*	*4*	
**CPZE**	*471.29 (306.62)*	*452.14 (254.61)*	*492.76 (359.02)*	*0.822^b^*
**Age of Psychosis Onset**	*24.64 (7.03)*	*25.30 (7.28)*	*23.91 (6.76)*	*0.380^b^*
**Duration of Illness**	*4.70 (5.72)*	*4.46 (6.30)*	*4.97 (5.09)*	*0.071^b^*
**PANSS Positive**	*16.90 (6.20)*	*12.00 (3.16)*	*22.39 (3.59)*	***<** **0.001^b^***
**PANSS ** **Negative**	*17.24 (5.77)*	*13.38 (3.47)*	*21.58 (4.65)*	***<** **0.001^b^***
**PANSS General**	*34.31 (8.37)*	*27.35 (3.\9)*	*42.12 (4.57)*	***<** **0.001^b^***
**PANSS Total**	*68.46 (18.28)*	*52.73 (5.46)*	*86.09 (8.97)*	***<** **0.001^b^***

*Note:* Continuous variables expressed as mean and standard deviation. Categorical variables expressed as number and percentage. p-Values for the comparisons between antipsychotic responders and non-responders were based on *t*-test (^a^), Mann-Whitney (^b^) and chi-squared (^c^) tests as appropriate. Bold indicate significant p values. There were no significant group differences in clinical or demographic characteristics other than in BMI and PANSS scores. n: number of subjects, BMI: body mass index, CPZE: chlorpromazine equivalent dose, PANSS: positive and negative syndrome Scale.

**Table 2:** Brain Glutamate and Plasma Cytokines.

Glutamate Levels	Responders (R)	Non-responders (NR)	*Test statistic*
*ACC*	− 0.14 (1.05)	0.08 (0.88)	F(1, 64) = 1.66, p = 0.202
Caudate	0.10 (0.94)	− 0.11 (0.90)	F(1, 61) = 0.34, p = 0.560
**Plasma Cytokines**			
IL-6	*0.79 (0.56)*	*1.39 (2.02)*	*F(1, 64) = 2.64, p = 0.109*
IL-8	*6.36 (2.44)*	*7.18 (3.66)*	*F(1, 64) = 2.30, p = 0.134*
IL-10	*0.38 (0.25)*	*0.47 (0.34)*	*F(1, 64) = 2.37, p = 0.128*
TNF-α	*3.24 (0.83)*	*3.30 (0.87)*	*F(1, 64) = 0.02, p = 0.888*
IFN-γ	*8.18 (5.91)*	*5.92 (5.18)*	*F(1, 64) = 2.79, p = 0.100*

Note: Variables expressed as mean and standard deviation. 1H-MRS metabolite concentration estimates are expressed as Z-scores, and cytokine concentrations are expressed as pg/mL. Analysis of variance was run to compare glutamate and plasma cytokine levels between groups. Statistical comparison on glutamate levels between R and TR was done correcting for age and sex, as in Egerton et al. (2021). Statistical comparisons on cytokine levels between R and NR were done on log transformed cytokine values, and corrected for age, sex, smoking status and BMI, as in Enache et al. (2021).

## Data Availability

Data governance frameworks are being put in place to make a fully anonymized version of the data available to the wider research community. To apply for access, contact JHM at james.maccabe@kcl.ac.uk
