# Supplementary material for: The association between peripheral inflammation, brain glutamate and antipsychotic response in Schizophrenia: Data from the STRATA collaboration
Source: Brain Behav Immun. Author manuscript; Available in PMC 2024 Feb 9. (PMC7615624; doi:10.1016/j.bbi.2023.05.005)
Supplement: Supplementary Material [file EMS193882-supplement-Supplementary_Material.pdf]

Supplementary Material

The Association between Peripheral Inflammation, Brain Glutamate and Antipsychotic Response in Schizophrenia: data from the STRATA collaboration

Sunniva Fenn-Moltu[a,b,c,†], Bill Deakin[d], Richard Drake[d], Oliver D Howes[a,e,f], Stephen M Lawrie[g], Shôn Lewis[h], Naghmeh Nikkheslat[i], James T R Walters[j], James H MacCabe[a,e], Valeria Mondelli[e,l,*], Alice Egerton[a,e,*]

a) Department of Psychosis Studies, Institute of Psychiatry, Psychology & Neuroscience, King’s College London, London, UK
b) King's College London, Institute of Psychiatry, Psychology and Neuroscience, Department of Forensic and Neurodevelopmental Sciences, London, UK
c) Centre for the Developing Brain, School of Biomedical Engineering & Imaging Sciences, King's College London, London, UK
d) Division of Neuroscience and Experimental Psychology, School of Biological Sciences, Faculty of Biology, Medicine and Health, University of Manchester, Manchester, UK
e) National Institute for Health Research (NIHR) Mental Health Biomedical Research Centre, South London and Maudsley NHS Foundation Trust, King's College London, UK
f) Psychiatric Imaging Group, MRC London Institute of Medical Sciences, Hammersmith Hospital, London, UK
g) Division of Psychiatry, University of Edinburgh, Edinburgh, UK
h) Division of Psychology and Mental Health, School of Biological Sciences, Faculty of Biology, Medicine and Health, University of Manchester, Manchester, UK
i) King's College London, Institute of Psychiatry, Psychology and Neuroscience, Department of Psychological Medicine, London, UK
j) MRC Centre for Neuropsychiatric Genetics and Genomics, Division of Psychological Medicine and Clinical Neurosciences, School of Medicine, Cardiff University, Cardiff, UK

* The authors equally contributed to the paper

† Corresponding author:

Ms Sunniva Fenn-Moltu
Institute of Psychiatry, Psychology and Neuroscience,
King’s College London, 16 De Crespigny Park, SE5 8AF, London, UK
sunniva.fenn-moltu@kcl.ac.uk

Supplementary Material

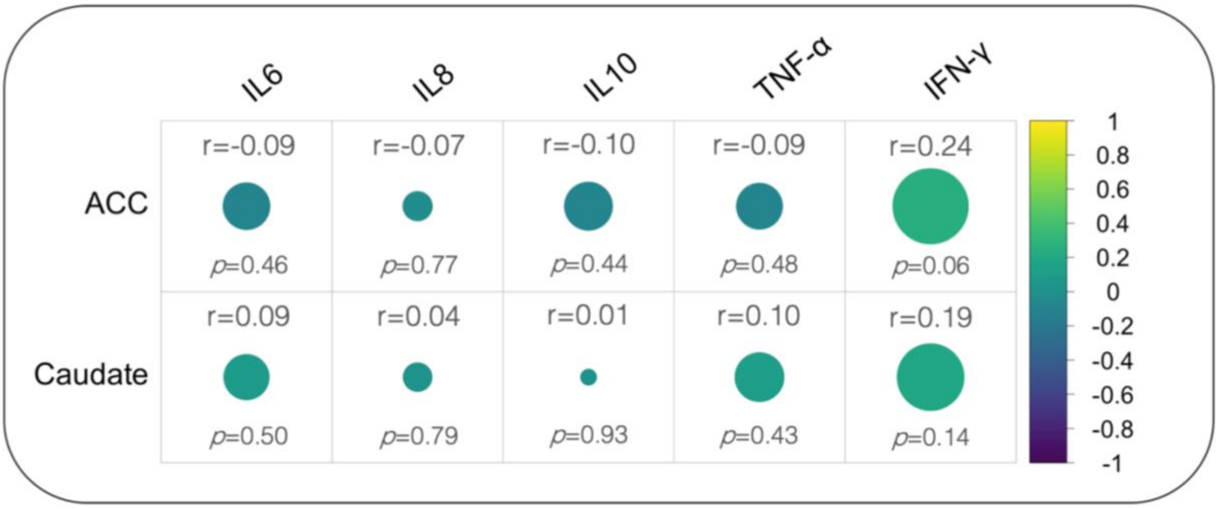

Supplementary Figure 1: The association between peripheral cytokine levels and Glx levels in the ACC and Caudate.

Supplementary Material

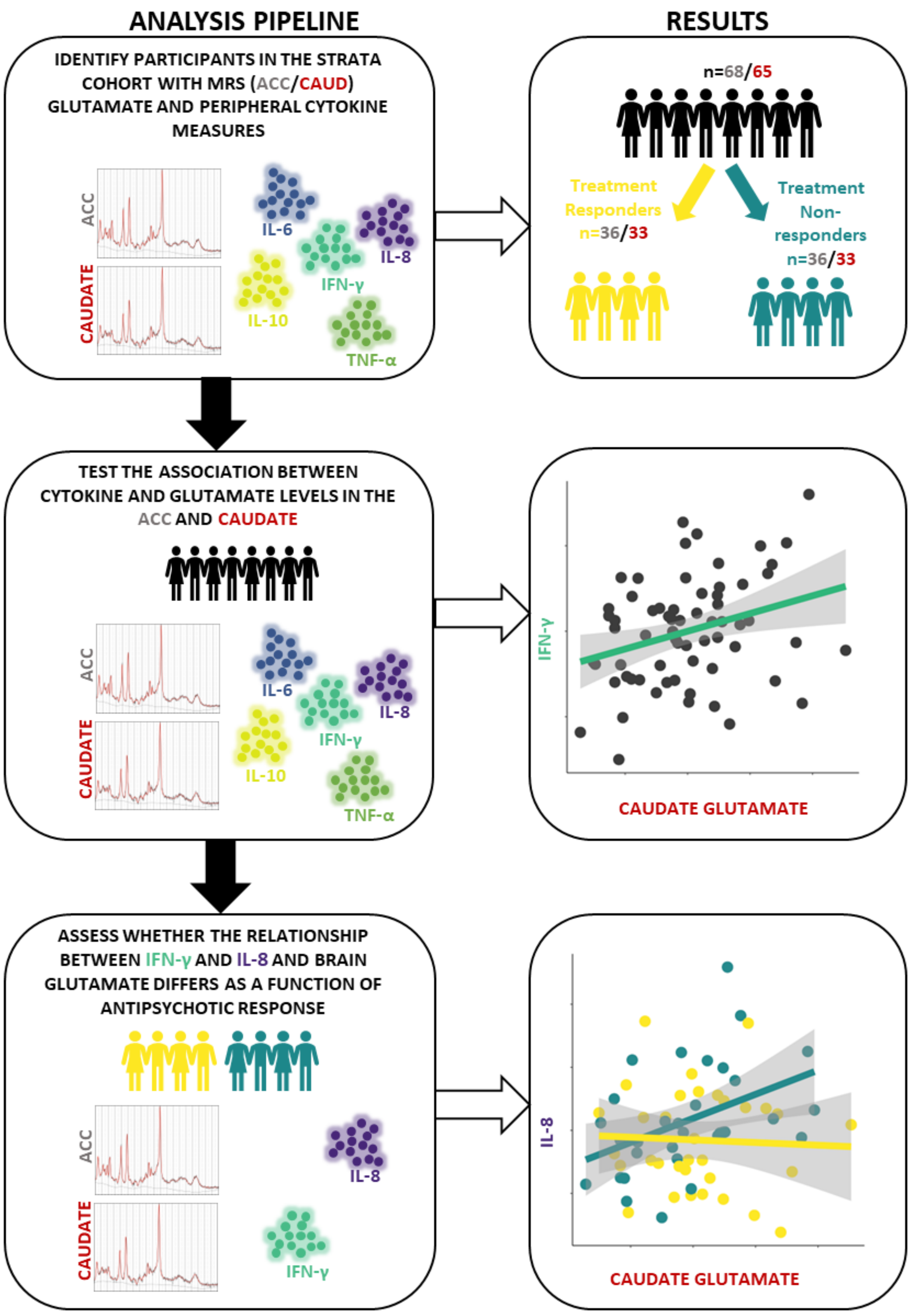

Supplementary Figure 2: An overview of the study design and main results.

Supplementary Material

Supplementary Table 1: Glx

Glx Levels	Total	Responders (R)	Non-responders (NR)	*Test statistic*
ACC	-0.01 (0.96)	-0.12 (1.07)	0.11 (0.82)	F(1, 64) = 1.29, p = 0.259
Caudate	-0.05 (1.00)	0.08 (0.97)	-0.19 (1.03)	F(1, 61) = 1.09, p = 0.300

Note: Variables expressed as mean and standard deviation. 1H-MRS Glx concentration estimates are expressed as Z-scores. Analysis of variance was run to compare Glx levels between groups correcting for age and sex, as in Egerton et al. (2021).
